# Dual roles of extracellular signal-regulated kinase (ERK) in quinoline compound BPIQ-induced apoptosis and anti-migration of human non-small cell lung cancer cells

**DOI:** 10.1186/s12935-017-0403-0

**Published:** 2017-03-07

**Authors:** Yao Fong, Chang-Yi Wu, Kuo-Feng Chang, Bing-Hung Chen, Wan-Ju Chou, Chih-Hua Tseng, Yen-Chun Chen, Hui-Min David Wang, Yeh-Long Chen, Chien-Chih Chiu

**Affiliations:** 1 Department of Thoracic Surgery, Chi-Mei Medical Center, Tainan, 710 Taiwan; 2 Department of Biological Sciences, National Sun Yat-sen University, Kaohsiung, 804 Taiwan; 3 Department of Biotechnology, Kaohsiung Medical University, Kaohsiung, 807 Taiwan; 4 The Institute of Biomedical Sciences, National Sun Yat-Sen University, Kaohsiung, 804 Taiwan; 5 School of Pharmacy, Kaohsiung Medical University, Kaohsiung, 807 Taiwan; 6 Graduate Institute of Biomedical Engineering, National Chung Hsing University, Taichung, 402 Taiwan; 7 Department of Medicinal and Applied Chemistry, Kaohsiung Medical University, Kaohsiung, 807 Taiwan; 8 Translational Research Center, Cancer Center and Department of Medical Research, Kaohsiung Medical University Hospital, Kaohsiung, 807 Taiwan; 9 Research Center for Environment Medicine, Kaohsiung Medical University, Kaohsiung, 807 Taiwan; 10 Graduate Institute of Medicine, College of Medicine, Kaohsiung Medical University, Kaohsiung, 807 Taiwan

**Keywords:** Indeno[1,2-*c*]quinoline, Quinoline, BPIQ, Lung cancer, ERK, MAPK, Apoptosis, Cellular migration

## Abstract

**Background:**

2,9-Bis[2-(pyrrolidin-1-yl)ethoxy]-6-{4-[2-(pyrrolidin-1-yl)ethoxy] phenyl}-11*H*-indeno[1,2-*c*]quinoline-11-one (BPIQ), is a synthetic quinoline analog. A previous study showed the anti-cancer potential of BPIQ through modulating mitochondrial-mediated apoptosis. However, the effect of BPIQ on cell migration, an index of cancer metastasis, has not yet been examined. Furthermore, among signal pathways involved in stresses, the members of the mitogen-activated protein kinase (MAPK) family are crucial for regulating the survival and migration of cells. In this study, the aim was to explore further the role of MAPK members, including JNK, p38 and extracellular signal-regulated kinase (ERK) in BPIQ-induced apoptosis and anti-migration of human non-small cell lung cancer (NSCLC) cells.

**Methods:**

Western Blot assay was performed for detecting the activation of MAPK members in NSCLC H1299 cells following BPIQ administration. Cellular proliferation was determined using a trypan blue exclusion assay. Cellular apoptosis was detected using flow cytometer-based Annexin V/propidium iodide dual staining. Cellular migration was determined using wound-healing assay and Boyden’s chamber assay. Zymography assay was performed for examining MMP-2 and -9 activities. The assessment of MAPK inhibition was performed for further validating the role of JNK, p38, and ERK in BPIQ-induced growth inhibition, apoptosis, and migration of NSCLC cells.

**Results:**

Western Blot assay showed that BPIQ treatment upregulates the phosphorylated levels of both MAPK proteins JNK and ERK. However, only ERK inhibitor rescues BPIQ-induced growth inhibition of NSCLC H1299 cells. The results of Annexin V assay further confirmed the pro-apoptotic role of ERK in BPIQ-induced cell death of H1299 cells. The results of wound healing and Boyden chamber assays showed that sub-IC_50_ (sub-lethal) concentrations of BPIQ cause a significant inhibition of migration in H1299 cells accompanied with downregulating the activity of MMP-2 and -9, the motility index of cancer cells. Inhibition of ERK significantly enhances BPIQ-induced anti-migration of H1299 cells.

**Conclusions:**

Our results suggest ERK may play dual roles in BPIQ-induced apoptosis and anti-migration, and it would be worthwhile further developing strategies for treating chemoresistant lung cancers through modulating ERK activity.

**Electronic supplementary material:**

The online version of this article (doi:10.1186/s12935-017-0403-0) contains supplementary material, which is available to authorized users.

## Background

Lung cancer is a leading malignancy in the world, especially in the Taiwan area [1]. Human non-small cell lung cancer (NSCLC) accounts for around 80% of total lung cancer cases [2]. The primary treatments for NSCLC patient are chemotherapeutics; however, the chemoresistance of NSCLC cells is frequently reported, resulting in poor prognosis and low survival rate of NSCLC patients [3]. Therefore, novel and improved chemotherapies for NSCLC cells are still being developed [3–7].

Compounds with a quinoline backbone have been shown to exert many bioactivities such as anti-autoimmune [8], anti-inflammatory [9] and anti-carcinogenic modalities [9–13]. For example, camptothecin (CPT), isolated from *Camptotheca acuminata*, exerts potent inhibitory activities against cancer cells, and two CPT derivatives topotecan and irinotecan are used for treating cancers clinically [14–16]. Accordingly, CPT-based derivatives are being developed for improving the anti-tumor activities [17, 18]. Our previous study demonstrated that

2,9-Bis[2-(pyrrolidin-1-yl)ethoxy]-6-{4-[2-(pyrrolidin-1-yl)ethoxy] phenyl}-11*H*-indeno[1,2-*c*]quinoline-11-one (BPIQ), a synthetic quinoline, exerts anti-growth and apoptosis-inducing potential against cancer cell lines including hepatocellular carcinoma cells [10, 12], non-small cell lung cancer (NSCLC) [19] and retinoblastoma cells [20]. Recently, our work further showed the BPIQ-induced apoptosis of cancer cells was mitochondrial-dependent [19].

Mitogen-activated protein kinase (MAPK) signaling pathways are involved in mediating processes of cell growth, survival, and death. There are three members of MAPK, JNK, p38 and ERK. Among MAPK members, JNK and p38 are activated in response to various intrinsic and extrinsic stresses [21, 22]. Additionally, activated p38 MAPK may induce apoptosis by phosphorylating or indirectly down-regulating pro-survival Bcl-2 family proteins under conditions such as cellular stress including ROS [23], DNA adducts [24] and starvation [25]. Previous studies indicate the mechanisms of many anti-cancer drugs are closely correlated with the stimulation of MAPK JNK and p38 [23, 26, 27].

On the contrary, the third member of MAPK, ERK is crucial for cell proliferation and survival and is activated by mitogenic stimuli, such as growth factors and cytokines [28]. Constitutive activation and overexpression of ERK are frequently observed in many cancer cells [29]. For example, more than 50% of acute myeloid leukemias and acute lymphocytic leukemias exert activated ERK pathways [30]. Additionally, the activated ERK pathway in lung cancer cells has also been reported [31].

Therefore, ERK targeting strategies against cancer have been used for treating cancer cells in vivo [32] and clinically [29].

On the contrary, ERK activation is not always correlated with pro-cellular survival. A recent study showed the interplays of ERK signaling and cell death, including apoptosis, autophagy, and senescence [33]. In a comparison of ERK targeting strategies, accumulating evidence demonstrated that activating ERK could take effect in cancer treatments [34–36]. Additionally, ERK signaling has also been involved in cell death induced by anti-cancer compounds including quercetin [37], betulinic acid [38] and miltefosine [39]. Besides, apoptosis induced by SU11274, a small molecule inhibitor of c-Met in NSCLC A549, has been associated with ERK-dependent p53 activation and Bcl-2 inactivation [36].

Contrarily, ERK has been shown to play an important role in cancer metastasis [40–42]. Likewise, our previous work also demonstrated that cardiotoxin III (CTX III), a basic polypeptide isolated from the venom of the Taiwan cobra (*Naja naja atra*) inhibits ERK-dependent migration and invasion of breast cancer cells MDA-MB-231 through down-regulating the signaling pathways of Src [43] and EGF/EGFR pathway [44].

In this study, we first examined whether the members of MAPK JNK, p38, and ERK involve in BPIQ-induced anti-NSCLC cells, and the dual roles of ERK in BPQI-induced anti-proliferation and anti-migration in NSCLC H1299 cells are also demonstrated. Furthermore, the possible mechanisms underlying ERK-mediated apoptosis of NSCLC cells induced by BPIQ are also discussed.

## Methods

### Preparation of BPIQ

BPIQ was synthesized described as previously published [10, 12]. BPIQ was freshly dissolved in DMSO (<0.01% final concentration) before assays.

### Reagents

DMEM and F12 medium, fetal bovine serum (FBS), trypan blue, penicillin G, and streptomycin were obtained from Invitrogen (Gaithersburg, MD, USA). Dimethyl sulphoxide (DMSO), ribonuclease A (RNase A), and propidium iodide (PI) were purchased from Sigma-Aldrich (St. Louis, MO, USA). Primary antibodies against JNK, p38 (sc-7149), p-p38 (Tyr^182^, sc-7973), ERK, p-ERK (Tyr^204^, sc-7976), COX-2, and β-actin (sc-7963) were obtained from Santa Cruz Biotechnology (Santa Cruz, CA, USA). Antibody against SP-1 (5407-S) was purchased from Epitomics. Antibody against p-JNK (Thr^183^/Tyr^185^, #07-175) was purchased from Millipore. Anti-rabbit, anti-goat and anti-mouse IgG peroxidase-conjugated secondary antibodies were purchased from Pierce (Rockford, IL, USA). Annexin V-FITC staining kit was purchased from Strong Biotech Co. Ltd. (Taipei, Taiwan).

### Cell culture

Human non-small cell lung cancer (NSCLC) cell line H1299 was obtained from the American Type Culture Collection (ATCC, Manassas, VA, USA). Cells were maintained in 1:1 ratio of DMEM: F-12 supplemented with 8% FBS, 2 mM glutamine, and the antibiotics (100 μg/ml streptomycin and 100 units/ml penicillin) at 37 °C in a humidified atmosphere of 5% CO_2_. All cells were tested to ensure the mycoplasma contamination-free using a PCR-based assay [45].

### Assessment of cell viability and morphological changes

Briefly, 1 × 10^5^ cells were seeded and treated with vehicle or various concentrations for 24 h. After incubation, the morphological changes of cells were observed by an inverted phase-contrast microscopy. For cell viability assessment, cells were trypsinized and stained with 0.2% trypan blue to count by Countess™ the automated cell counter (Invitrogen, Carlsbad, CA, USA).

### Western Blot analysis

Western Blot assay was conducted according to a previously published article [46]. In brief, cells were harvested and lysed. A total of 20 μg protein lysate was resolved by 10% SDS-polyacrylamide gel electrophoresis (SDS-PAGE) and electro-transferred. The nitrocellulose membrane was blocked with 5% non-fat milk and incubated with primary and secondary antibodies sequentially. The signals for specific proteins were detected using a chemiluminescence-based ECL™ detection kit (Amersham Piscataway, NJ, USA).

### Apoptosis assessment

The Annexin V/PI double staining assay recognizes the externalization of phosphatidylserine (PS) on the cell membrane, a hallmark of apoptotic cells. In brief, 5 × 10^5^ cells were seeded on a 100-mm petri dish and treated with BPIQ alone or 2 h pre-treatment of an ERK inhibitor PD98059 for 24 h respectively. Cells were suspended with trypsin, harvested and stained with Annexin V/PI. Afterward, the cells were analyzed by a flow cytometer (FACS Calibur; Becton–Dickinson, Mountain View, CA, USA).

### Assessment of cell migration

3 × 10^5^ H1299 cells were seeded into a 12-well plate, then treated with indicated concentrations of BPIQ and a 1-mm wide wound area was created using a 200 µl plastic tip. After 16 h incubation, the wound areas were photographed and automatically calculated using the free software tool “TScratch” [47].

### Boyden’s chamber assay

The invasion of cancer cells was performed by a 24-well transwell unit with Matrigel™ (Greiner Bio-One, Frickenhausen, Germany) coated on the upper side of polycarbonate filters into 8 μm filter pore size transwell inserts. The lower well was injected with 800 µl medium containing 10% FBS, without or with indicated concentrations of BPIQ. 1 × 10^5^ H1299 cells was resuspended in 200 µl of serum-free medium were seeded onto a transwell insert and allowed to invade for 16 h. Non-invaded cells on the upper part of the membrane were removed. Cells on the bottom surface of the filters were fixed with 4% paraformaldehyde, stained with Giemsa (Merck), and counted under a microscope. Each experiment was done in triplicate, and the results from three independent experiments were expressed as mean ± SD.

### Assessment of MAPK inhibitors

To determine the effects of MAPK ERK, p38, and JNK on BPIQ-induced apoptosis, three specific inhibitors (50 μM), PD98059 (Sigma) for ERK, SB203580 (Sigma) for p38 and SP600125 (Sigma) for JNK, were dissolved in DMSO respectively. The assessment has been described previously [46]. In brief, seeded cells were pre-treated with MAPK inhibitors for 2 h respectively. Afterwards, cells were administrated with 24 h treatment of BPIQ for cell proliferation assay and annexin V staining, and 16 h for Boyden’s chamber assay.

### Gelatin zymography

The gelatin zymography [48] was performed for detecting the gelatinases MMP-2 and -9 using 10% polyacrylamide gels contained 0.1% gelatin. After electrophoresis, SDS was replaced using 2.5% Triton X-100, followed by incubation in a Tris-based buffer containing NaCl, CaCl_2_, and ZnCl_2_ at 37 °C overnight. The gel was then stained with Coomassie Brilliant Blue R-250, and gelatinase activity was detected as unstained gelatin-degradation zones within the gel. The signals were analyzed using Gel-Pro 3.0 software (Media Cybernetics, Silver Spring, MD, USA).

### Statistical analysis

Differences between cells treated with vehicle were analyzed in at least triplicate. The statistical differences were analyzed by one-way analysis of variance (ANOVA) using SigmaPlot v12 (Systat Software Inc.) and *p < 0.05 vehicle vs. BPIQ treatment was considered statistically significant.

## Results

### Cellular morphology assessment following BPIQ treatment

**Fig. 1:**
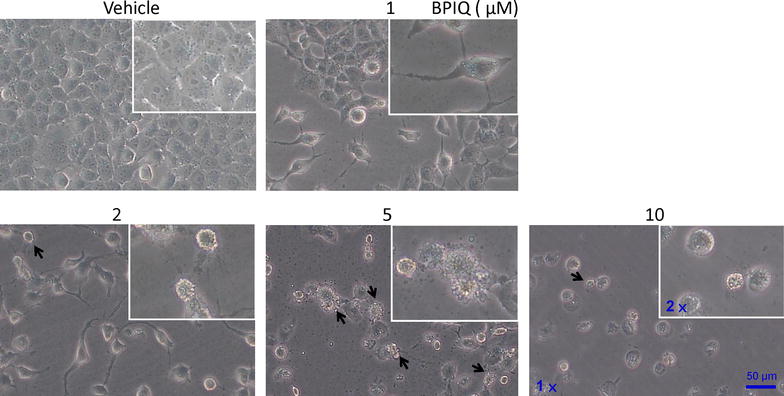
Effect of BPIQ on cellular growth and morphological changes of lung cancer cells. H1299 cells were seeded and treated with indicated concentrations of BPIQ for 24 h. The *arrows* indicate the blebbing membrane of cells, a hallmark of cellular apoptosis. Magnification: 100 and 200×

H1299 cells were treated with indicated concentration (from 1 to 10 μM) of BPIQ to assess the effect of BPIQ on cellular growth and cellular morphological changes. As shown in Fig. 1, the decrease of the cell population and the significant morphological changes, including the cell shrinkage, blebbing membrane and the formation of apoptotic bodies was observed at 24 h following BPIQ treatment (the results of 6 and 12 h treatment are shown in Additional file 1). A marked increase of population rounding cells and apoptotic bodies appeared when the used concentrations began at 5 μM BPIQ, suggesting the dose-dependent effect of BPIQ.

### BPIQ induces the activation of MAPK proteins in NSCLC cells

**Fig. 2:**
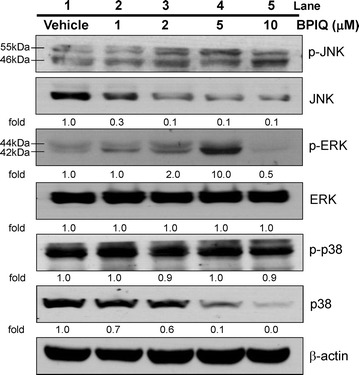
Activation of MAPK family in BPIQ-treated lung cancer cells. The inhibitor assay was performed to determine the role of MAPK family in BPIQ-induced apoptosis of lung cancer cells. Briefly, cells were seeded and treated with indicated concentrations of BPIQ for 24 h respectively. 20 μg protein lysates were resolved by 10% SDS-PAGE and Western Blot assay for detecting the activation of MAKP members JNK, p38, and ERK. β-actin, an internal control for equal loading. Each experiment blot is representative of three independent experiments

As shown in Fig. 2, the results of the Western Blot assay showed that the treatments with BPIQ dramatically increased the phosphorylation of ERK and JNK, whereas no significant changes in p38 activation were observed. Interestingly, total protein levels of both JNK and p38 were dramatically decreased.

### ERK blockade rescues BPIQ-induced anti-proliferation of NSCLC cells

**Fig. 3:**
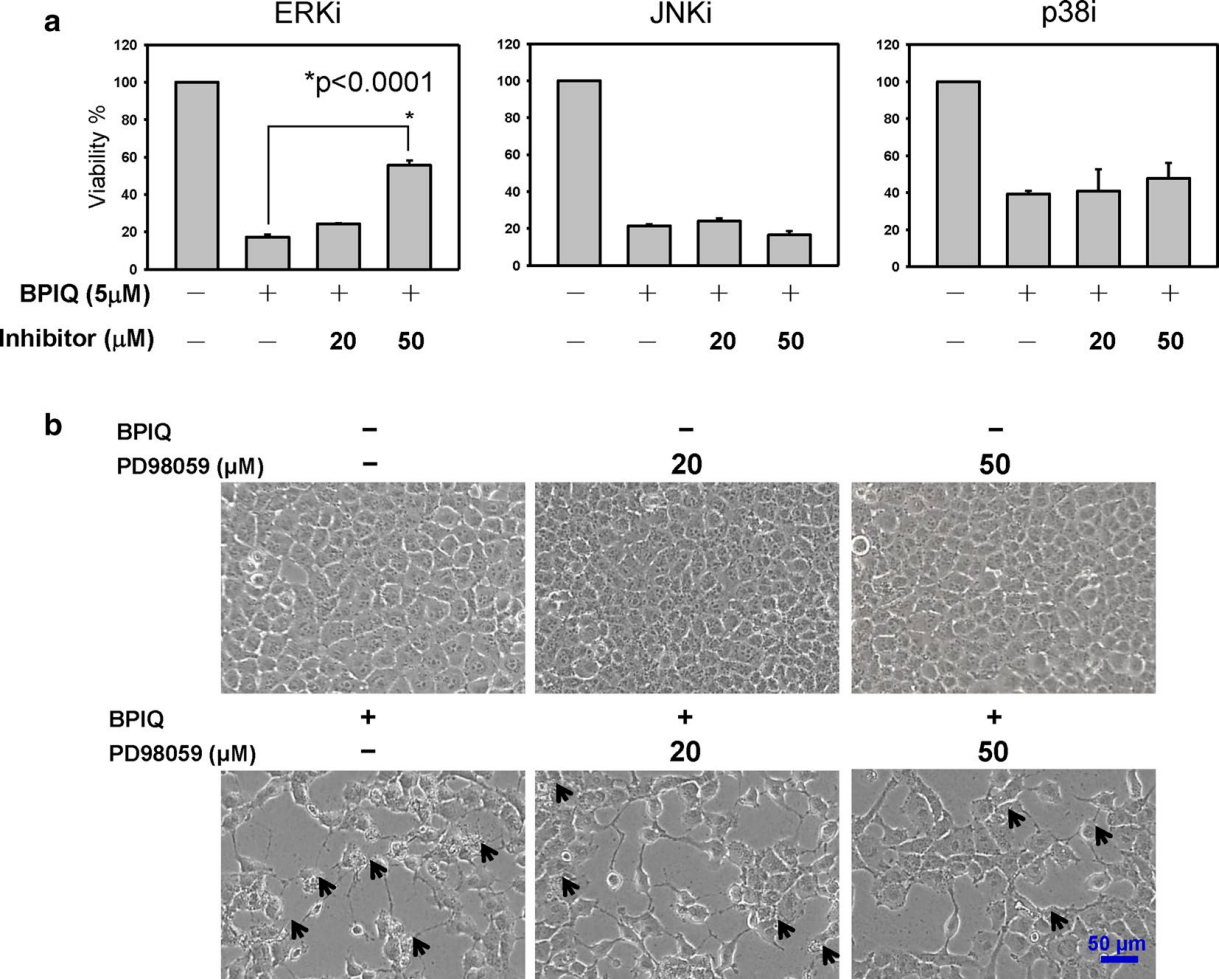
The effect of MAPK inhibitors on BPIQ-induced anti-proliferation of lung cancer cells. H1299 cells were subject to treatment with BPIQ alone or MAPK specific inhibitors for 2 h prior to BPIQ administration for 24 h. The result of cellular survival assay is represented. Specific MAPK inhibitors, PD98059 for ERK, SP600125 for JNK, and SB203580 for p38 before BPIQ administration respectively. **a** Data were statistically analyzed with Student’s *t* test (*p < 0.05 BPIQ vs. BPIQ with inhibitor pre-treatments). **b** The ERK inhibitor rescues the decrease in cell number and morphological changes induced by BPIQ in H1299 cells. Magnification: 100×

To examine whether the MAPK family plays a role in BPIQ-induced anti-proliferation and growth of NSCLC H1299 cells, the specific inhibitors of the MAPK family including PD98059 for ERK, SP600125 for JNK and SB200358 for p38 were pretreated prior to the BPIQ administration. As shown in Fig. 3a, the results of the proliferation assay demonstrated that the inhibition of ERK significantly rescues the proliferation inhibition of H1299 cells induced by BPIQ treatment. Likewise, ERK blockade partially rescues the morphological changes induced by BPIQ, including cell rounding and membrane blebbing compared to BPIQ treatment alone (Fig. 3b). These results suggest the anti-survival role of ERK in BPIQ-induced anti-proliferation in NSCLC cells.

### ERK blockade rescues BPIQ-induced apoptotic death of NSCLC cells

**Fig. 4:**
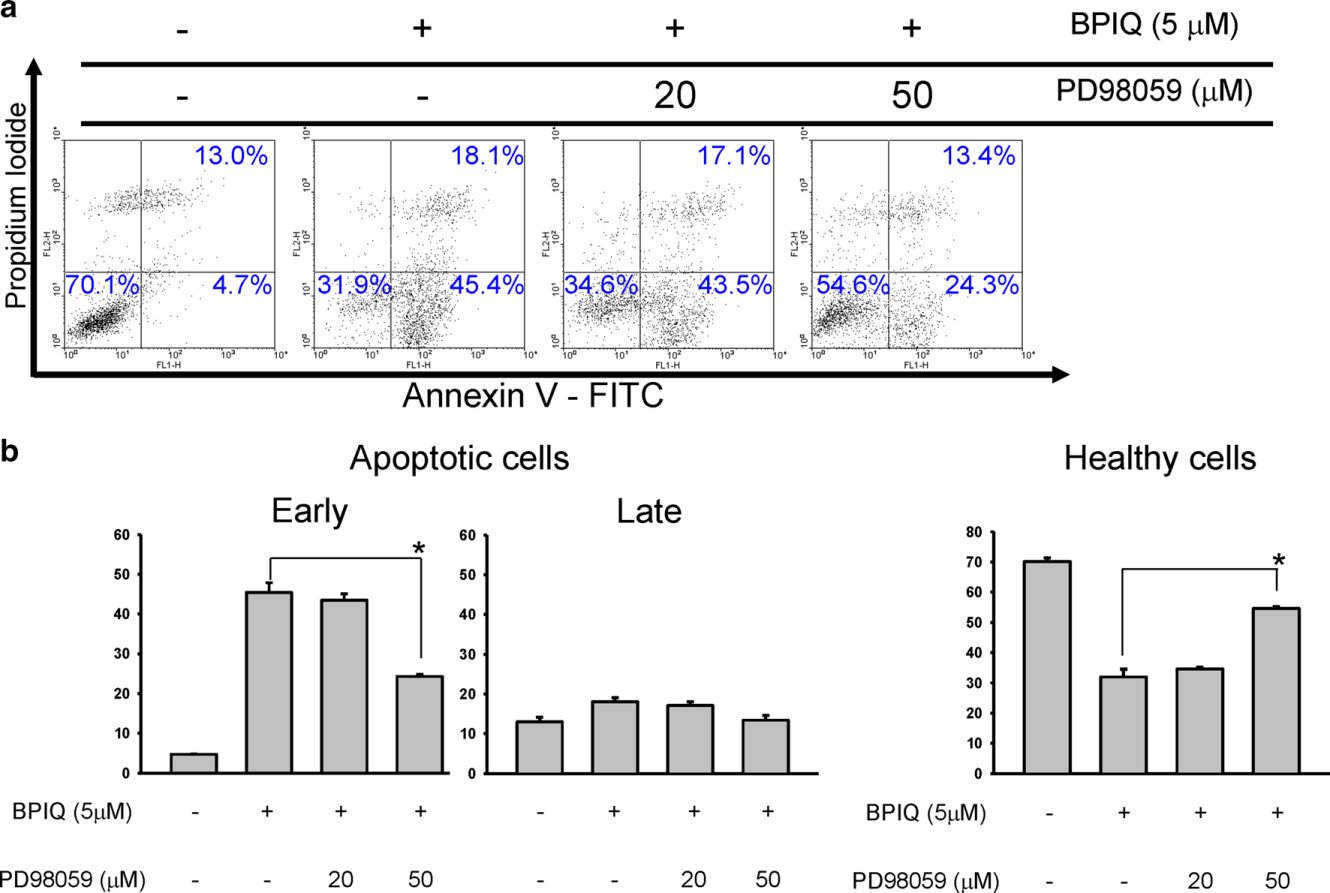
ERK blockade rescues BPIQ-induced apoptosis of NSCLC cells. Cells were pre-incubated for 2 h with the following specific MAPK ERK inhibitors, PD98059 prior to BPIQ administration (see “Methods” section). Subsequently, the apoptotic populations induced by BPIQ were determined using flow cytometer-based Annexin V/PI staining. **a** Results of Annexin V/PI staining and **b** the quantitative analysis. Data are presented as mean ± SD. of at least three experiments independently. The results were analyzed with the statistical approach Student’s *t*-test (*p < 0.05 BPIQ vs. BPIQ with inhibitors pre-treated)

To further confirm the role of ERK in BPIQ-induced anti-NSCLC effect, we determine the effects of ERK on BPIQ-induced cell death. As shown in Fig. 4a, b, the result of Annexin V/PI double staining showed that inhibiting ERK activity rescued BPIQ-induced apoptosis of H1299, especially in the early stage of apoptosis. The percent healthy cells were elevated from 31.9 to 54.6% following pre-treatment with ERK inhibitor (Fig. 4b). These results are consistent with the results of Fig. 3, indicating a pro-apoptotic role of MAPK ERK in BPIQ-induced apoptosis in human NSCLC tumor cells.

### BPIQ attenuates the migration of NSCLC cells

**Fig. 5:**
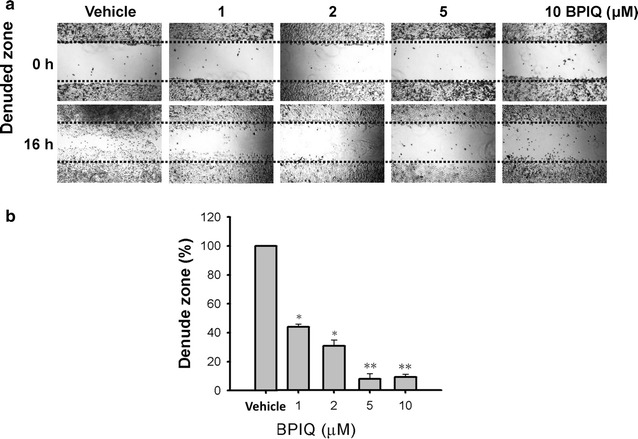
The effect of BPIQ on the cellular migration of NSCLC cells. **a** 5 × 10^5^ H1299 cells (confluent culture) were seeded in a 12-well plate, and cells were scraped to create a 1-mm wide wound area. Cells were treated with indicated concentrations (from 0 to 10 μM) of BPIQ for 16 h. Afterward, the wound areas were photographed using an inverted phase-contrast microscopy. **b** Quantitative analysis of **a**. **p < 0.05 and **p < 0.001 against the vehicle respectively. Magnification: 100×

Figure 5 shows that the migration ability of H1299 lung cancer cells was dramatically inhibited by BPIQ, and reveals that the migration ability of H1299 cells treated with various BPIQ concentrations at 0, 1, 2, 5 and 10 μM was 100, 43.96 ± 1.78, 30.76 ± 4.01, 7.87 ± 3.58 and 9.17 ± 1.84% (n = 3) respectively. These results indicate that BPIQ-induced anti-migration of NSCLC H1299 cells is dose-responsive.

### BPIQ inhibits the cellular invasion of NSCLC cells

**Fig. 6:**
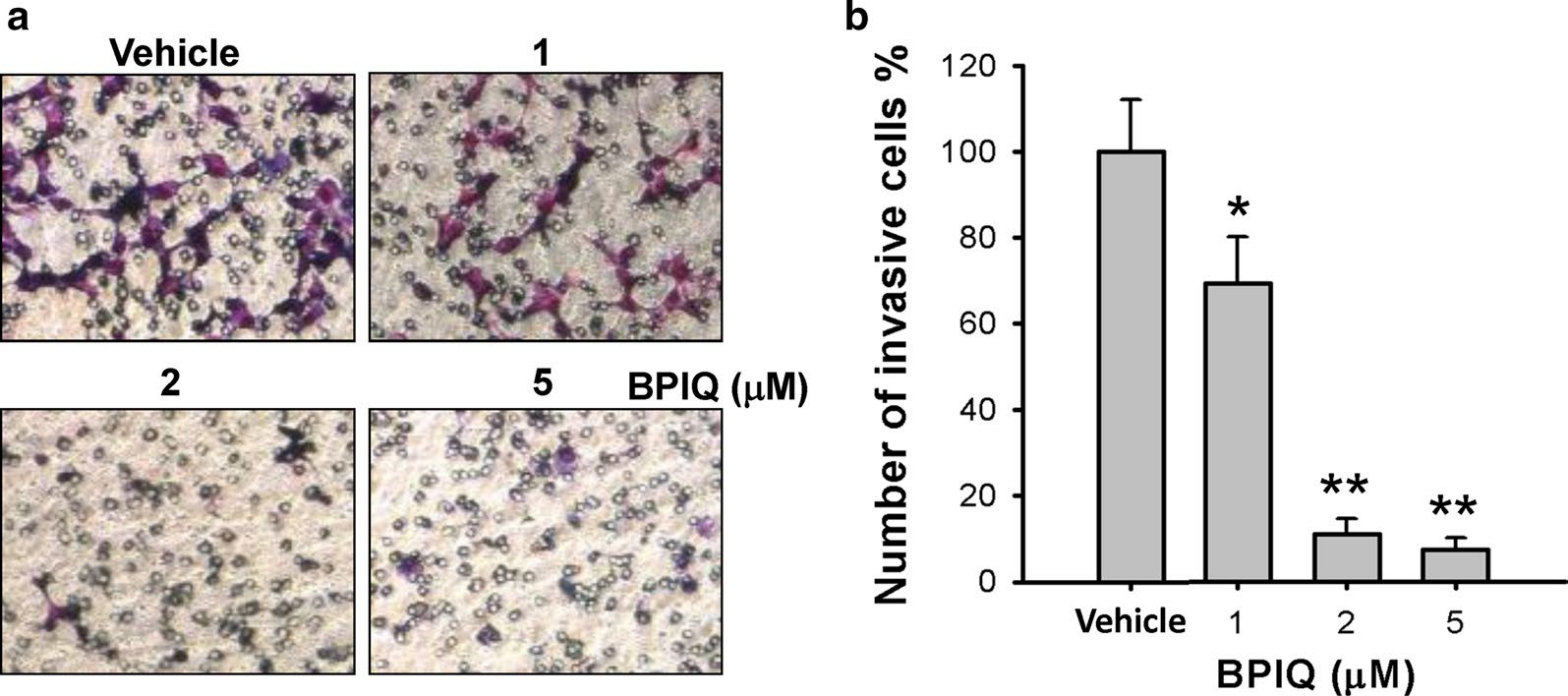
The effect of BPIQ on the invasion of NSCLC cells. The Boyden’s chamber assay was performed to examine the effect of BPIQ on cellular invasion, **a** cells were treated with indicated concentrations of BPIQ for 16 h and invaded cells were analyzed using a modified Boyden’s chamber. Cells in serum-free DMEM-F12 were added to the upper chamber and allowed to migrate through 8-μm porous membrane toward a lower chamber in medium with serum. **b** The cellular motility was quantified by counting the number of cells that invaded to the undersides of the membrane under a microscopy (magnification: 100×). The results are presented as mean ± SD of triplicate experiments, *p < 0.05 and **p < 0.01 against vehicle respectively

The invasion ability of H1299 cells was assessed by Boyden’s chamber migration assay. As shown in Fig. 6, BPIQ inhibits the mobility of H1299 cells in a non-cytotoxic dose (less than 2 μM). Figure 6 revealed that the invasion ability of H1299 cells treated with various BPIQ concentrations at 0, 1, 2 and 5 μM was 100 ± 12.25, 69.12 ± 11.01, 10.84 ± 3.75 and 7.36 ± 2.67% (n = 3) respectively. These results indicate that sub-IC_50_ dose (below 2 μM) of BPIQ is effective to suppress the invasion of H1299 lung cancer cells.

### BPIQ down-regulates the expression of migration-associated proteins

**Fig. 7:**
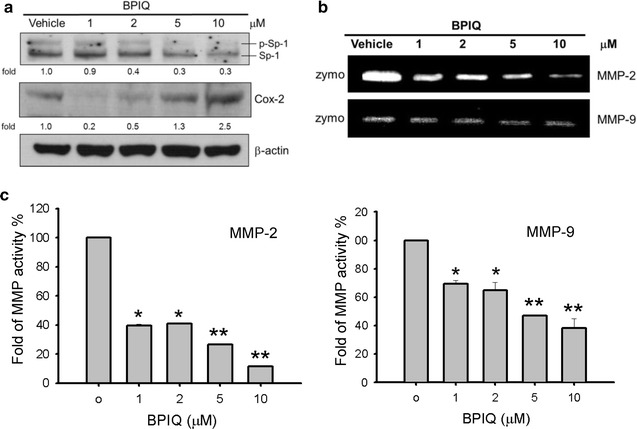
The regulation of cellular migration-associated proteins by BPIQ treatment. H1299 cells were subject to the treatment with indicated concentrations of BPIQ for 24 h. **a** Western Blot showed that sub-IC_50_ of BPIQ inhibits the phosphorylation of SP-1 and decreases the protein level of COX-2. β-actin as an internal control. Each blot is representative of three independent experiments. **b** The zymography assay. These results showed that BPIQ attenuates the activities of MMP enzymes especially MMP-2 in a dose-responsive manner. **c** The quantitative analysis of **b** indicates that sub-IC_50_ of BPIQ (less than 2 μM) significant inhibits MMPs activities. *p < 0.05 and **p < 0.01 against vehicle respectively

The transcription factor specificity protein 1 (SP-1) has been shown to play a critical role in both proliferation and migration of cells [49]. The overexpression or constitutive activation of SP-1 has shown to be involved in tumor development and metastasis of cancer cells, including brain tumor astroglioma and gastric cancer and lung cancer [50–52]. SP-1 was also reported to promote invasion and migration of cancer cells by upregulating expression of the metastasis-associated proteins integrin α5 and cadherin-11[53]. Regarding the migration, the target genes of SP-1 including cyclooxygenase-2 (COX-2), MMP-2 and MMP-9 were closely correlated with cellular migration [54–56]. As shown in Fig. 7a, after treatments with vehicle or indicated concentrations of BPIQ, the phosphorylation of SP-1 and the protein level of SP-1 downstream target COX-2 was significantly decreased at a sub-IC_50_ dose of BPIQ treatment. Likewise, the results of gelatin zymography assay showed that BPIQ attenuates the activities of MMP-2 and -9, especially MMP-2 in a dose-dependent manner (Fig. 7b, c). These above results suggested that sub-IC_50_ dose of BPIQ significantly inhibits the migration of H1299 cells through modulating the expression and activation of a panel of migration-associated proteins such as SP-1 and COX and the downregulation of MMP-2 and -9 activities

### The role of ERK in sub-IC_50_ BPIO-induced anti-migration of H1299 cells

**Fig. 8:**
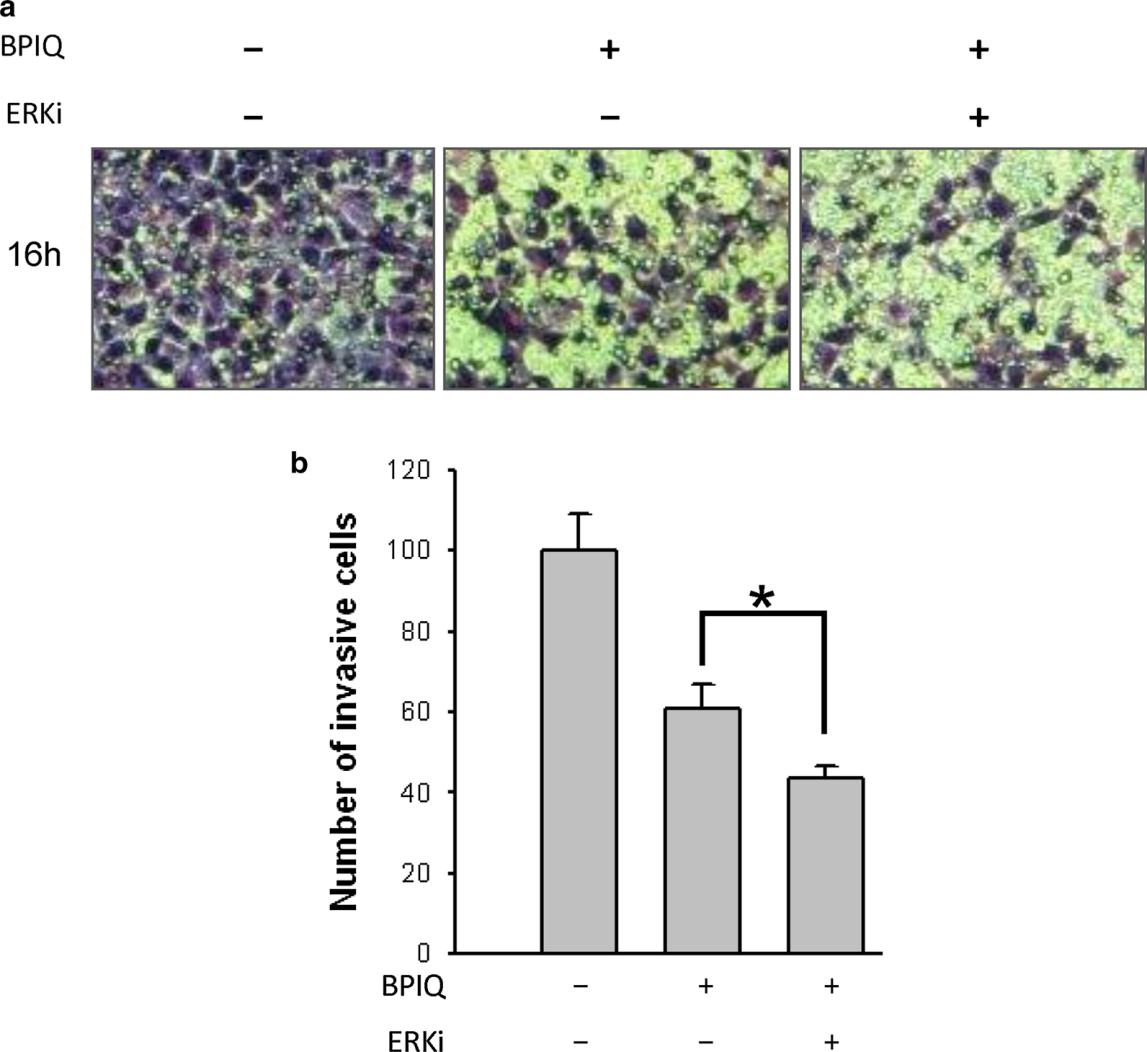
ERK blockade enhances BPIQ-induced anti-migration of NSCLC cells. **a** The cellular mobility was determined using the Boyden’s invasion assay. H1299 cells were treated with vehicle, 0.2 μM BPIQ alone or 0.2 μM BPIQ with 50 μM ERK inhibitor PD098059. **b** The quantitative results of (**a**). *p < 0.05 for BPIQ vs. BPIQ with pretreatment of ERK inhibitors

Besides cell survival, ERK also has been shown to play a major role in cellular migration. For example, ERK expression promotes the migration of melanoma cells. Otherwise, the attenuation of ERK signaling by depleting epidermal growth factor (EGF) inhibits anti-migration of cancer cells [57, 58]. Figure 8 shows that the invasion ability of H1299 cells treated with vehicle, BPIQ alone and the BPIQ with ERK inhibitor pretreatment was 100 ± 5.18, 57.98 ± 7.36, 61.64 ± 3.65% (n = 3) respectively. This result shows that ERK inhibitor additively enhances 20% of BPIQ-induced anti-invasion in NSCLC H1299 cells, suggesting the pro-migration role of ERK in H1299 cells.

## Discussion

Mitogen-activated protein kinase (MAPK) signaling pathways are involved in mediating processes of cell growth, survival and death [21, 22]. MAPK members p38 and JNK pathways have been reported to induce apoptosis under various cellular stresses [59, 60]. Therefore, many anti-cancer drugs are designed for stimulating JNK and p38-mediated apoptosis of cancer cells such as breast cancer [26], colon cancer [23] and lung cancer cells [27]. However, the role of MAPK members in anti-cancer drugs-induced apoptosis may depend on cell types and the stimuli, and studies suggesting the pro-survival role of p38 MAPK in cancer cells toward anti-cancer drugs were also reported [61, 62]. For example, Bruzzese’s work reported that the activation of p38 MAPK was associated with the resistance of prostate cancer (PCa) and multiple myeloma (MM) cells towards zoledronic acid (ZOL), a nitrogen-containing bisphosphonate. In addition, panobinostat, a histone deacetylase inhibitor, was shown to render both PCa and MM sensitive to ZOL by inhibiting the activity of p38 MAPK [61]. Furthermore, DU145R80, a ZOL-resistant prostate cancer cell line, expresses p38 MAPK-dependent survival pathway accompanied with an enhanced potential for epithelial-mesenchymal transition (EMT) and the increased expression of metalloproteases MMP-2/-9 compared to its parental cell line, suggesting the essential role of p38 MAPK in acquiring chemoresistance of prostate cancer cells [62].

Despite the potential of BPIQ on anti-proliferation of cancer cells, the role of MAPK in BPIQ-induced growth inhibition is not clear. To further clarify the mechanism underlying MAPK-induced apoptosis and anti-proliferation induced by BPIQ. The cellular and molecular parameters about BPIQ-induced apoptosis were studied using three NSCLC tumor cells H1299. The results of Western Blot showed the activation of two MAPK members JNK and ERK was detected after BPIQ treatment (Fig. 2).

Therefore, we determined whether JNK or ERK plays a role in BPIQ-induced apoptosis and anti-proliferation and performed the MAPK inhibitor assays. The inhibitor assay showed that blockade of ERK activity significantly rescued BPIQ-induced anti-proliferation (Fig. 3) and apoptotic cell death (Fig. 4) of NSCLC tumor cells. Regarding the correlation between ERK signaling and the process of cell death, ERK is thought to be critical for cell survival and mediating a survival response that counteracts with cell death and its activation being frequently observed in cancer cells [29]. For example, Caraglia’s work demonstrated that the combination of ZOL and R115777, a non-peptidomimetic farnesyl transferase inhibitor, exerted a synergistic effect on apoptosis induction in cell lines of prostate adenocarcinoma through dramatically attenuating Ras signaling and its downstream targets, namely the ERK and Akt survival pathways [63]. Likewise, the combination of Simvastatin, an HMG–coenzyme A reductase inhibitor, and R115777 (Tipifarnib) exerted a cooperative effect on anti-proliferation and apoptosis induction of two NSCLC cell lines GLC-82 (adenocarcinoma) and CALU-1 (squamous-carcinoma) by inhibiting Ras/Raf/MEK/ERK signaling [64].

On the contrary, many studies also showed the correlations of ERK signaling and stimulating the process of cell deaths [33, 65, 66]. ERK pathways may induce apoptosis through promoting caspase-8 signaling and the activation, or potentiating the activation of death receptors by increasing the level of death ligands such as TNFα or FasL, or death receptors such as Fas, DR4 or DR5. For example, llimaquinone, an anti-cancer agent, was found to upregulate the expression of death receptor DR-4/-5 through ERK activation in colon cancer cell lines HCT116 and HT-29 [67].

Additionally, ERK activity was reported to promote the induction of FADD, an adaptor of caspase-8 for death receptors. Furthermore, an antibiotic fluoroquinolone was reported to induce the apoptosis of pancreatic cancer through ERK-dependent mitochondrial pathways, including the proteolytic activation of caspase-9, the loss of mitochondrial membrane potential, and the up-regulated expression of pro-apoptotic Bax and Bak [68].

Wang’s work suggested that ERK activation may contribute to activin A-induced apoptosis of NSCLC cells A549 [69]. Similarly, piperlongumine, a bioactive compound isolated from large peppers, was reported to induce the cell death of colon cancer HT-29 cells through MEK-ERK signaling [70]. Furthermore, recent studies also showed that antitumor compounds, such as quercetin [37], betulinic acid [38], miltefosine [39] induce ERK-dependent apoptosis. Besides, a cytotoxin VacA secreted by *Helicobacter pylori*, a Gram-negative bacterium, was reported to induce apoptosis of gastric cancer cells. [34]. Likewise, SU11274, a small molecule inhibitor of c-Met was reported to induce apoptosis of lung cancer cells A549 through ERK-p53 and ERK-mediated Bcl-2 phosphorylation [36], indicating the pro-apoptotic role of ERK and its applications in cancer treatment. Accordingly, the activation of ERK-dependent apoptotic signaling may be a promising treatment for chemoresistant cancer cells especially those that overexpress ERK.

Many anti-cancer drugs have been shown to exert multi-effects against cancer cells. For example, curcumin, a diferuloylmethane, induces both apoptosis and anti-migration of human medulloblastoma cells [71]. We therefore examined whether BPIQ exerts anti-cancer activities beyond anti-growth and the induction of apoptosis. Both the wound healing and Boyden’s chamber assays demonstrate that sub-IC_50_ of BPIQ (below 2 μM) significantly inhibits the cellular mobility of H1299 cells. We next tried to depict the mechanism underlying BPIQ-induced anti-migration in lung cancer. The upregulation of pro-inflammatory COX-2 expression, MMP-2 and -9 have been reported to be associated with the progression of malignant tumors [72, 73]. Moreover, the expressions of MMP-2 and MMP-9 are regulated by SP-1 [54, 74].

As shown in Fig. 7, the inactivation of migration-associated factor SP-1 following BPIQ treatment was also observed. Furthermore, the protein level of COX-2 and the activity of MMP-9 and -2 were also decreased. In cell signal pathways, the phosphorylation of many signaling proteins is thought to be dynamic and transient [75]. Our previous work showed that magnolol, a compound isolated from the herbal plant Magnolia induced apoptosis of NSCLC A549 cells through upregulating the activity of MAPK p38 and JNK. Both the phosphorylations of MAPK p38 and JNK were increased following magnolol treatment in a dose-responsive manner, whereas the dramatic decrease of phosphorylation was observed at the highest dose [76]. Likewise, metformin which exert anticancer activities, was reported to increase a dose-responsive phosphorylation of ERK but decrease at the highest dosage in neuroendocrine tumor cells BON1 and NCI-H727 [77]. Similarly, the results of Western Blot assay showed that the phosphorylation of ERK following BPIQ treatments (from 1 to 5 μM) for 24 h was dose-responsive. We therefore suggested that the highest concentration (10 μM) of BPIQ may cause the phosphorylation of ERK earlier than 24 h of treatment and return to the un-phosphorylated or hypo-phosphorylated status after 24 h.

These above observations suggest that the orchestrate signaling modulated by BPIQ eventually led to the inhibition of cellular migration.

Accumulating evidence showed that the cell migration was promoted by MPAK ERK, and the inhibition of cell migration was often accompanied by attenuated activities of ERK in cancer cells [57, 58]. Consistently, the results of our study indicate the pro-migration role of ERK and ERK blockade enhances the inhibitory effect of BPIQ on migration and invasion of H1299 cells (Fig. 8a, b). Accordingly, it will be an advantage to inhibit ERK activation combining BPIQ treatment against cancer cells in further study. A low- or non-cytotoxic dose of BPIQ combining the inhibitors of ERK such as PD98059 may be anti-metastatic or chemopreventive strategies for NSCLC treatment in future.

## Conclusions

**Fig. 9:**
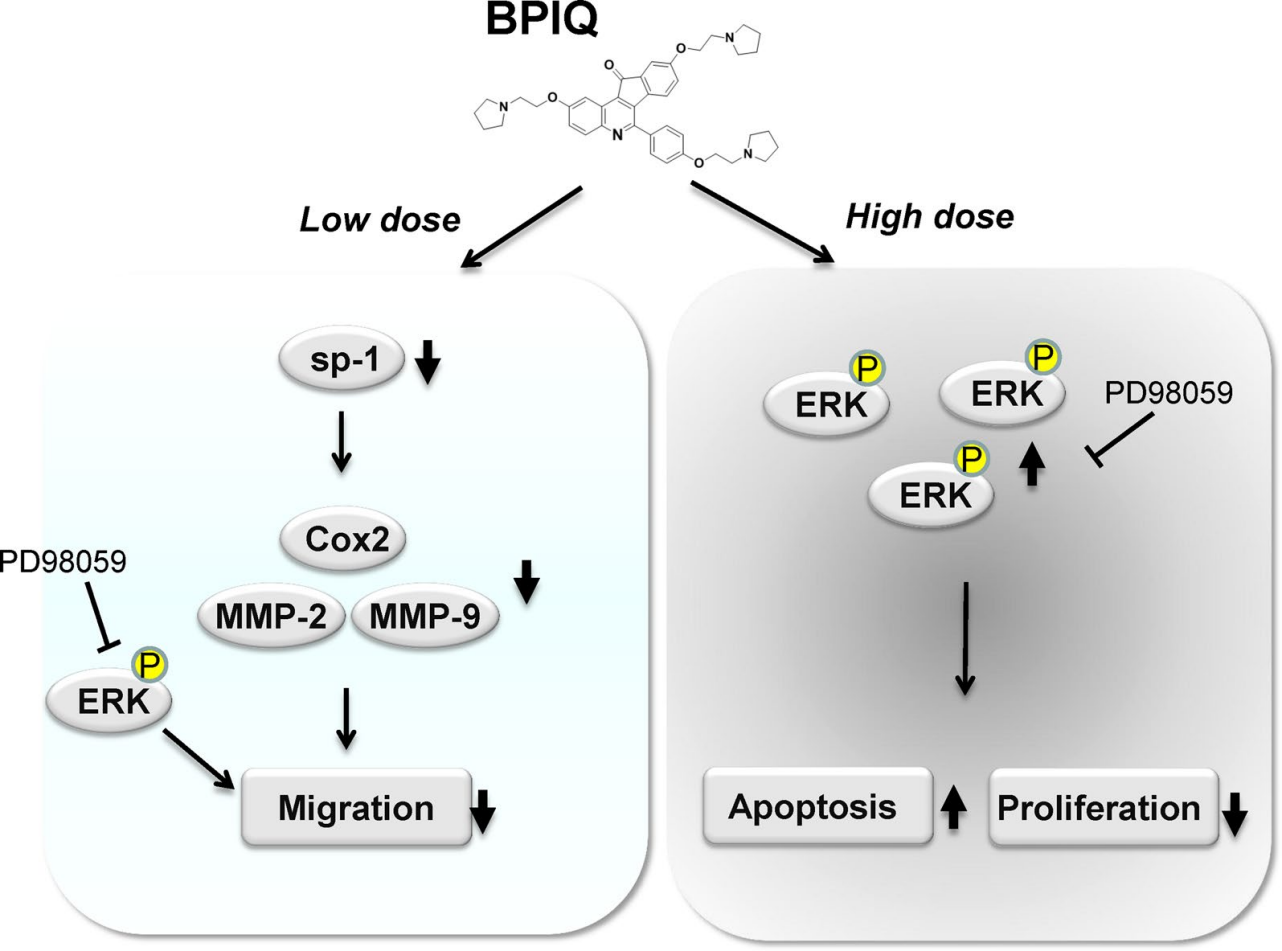
The possible model for the roles of ERK in BPIQ-induced apoptosis and anti-migration of lung cancer cells. Sustainable ERK or overexpression are thought to promote cellular proliferation and survival in cancer cells. However, the activation of ERK may involve sensitizing cancer cells to BPIQ, eventually induce the apoptosis of NSCLC cells. It will benefit regulation of ERK-dependent apoptosis of cancer cells which highly express ERK. On the contrary, low dose (sub-IC_50_) of BPIQ exerts the anti-migration of NSCLC cells through downregulating the activation of SP-1 transcription factor and its downstream such as COX and MMP activity. In future, low dose of BPIQ combining ERK inhibitors such as PD98059 may be a promising strategy for chemoprevention or anti-metastasis of NSCLC cells

Our present result suggests that the activation of ERK signaling, at least partially, was responsible for BPIQ-induced anti-proliferation and apoptosis of NSCLC tumor cells. On the contrary, a sub-lethal dose of BPIQ attenuates cellular migration of NSCLC cells through inhibiting ERK activity, suggesting the dual roles of ERK in BPIQ-induced apoptosis and anti-migration of NSCLC cells. The results of our study may benefit apoptosis induction and chemoprevention of lung cancer cells through ERK signaling (Fig. 9).

## Additional file

**Additional file 1.** The effect of BPIQ on a time course of morphological changes of lung cancer cells. H1299 cells were treated with indicated concentrations of BPIQ for 6 and 12 h respectively. No significant changes of cellular morphology were observed. Magnification: 100×.

## Abbreviations

BPIQ: 2,9-Bis[2-(pyrrolidin-1-yl)ethoxy]-6-{4-[2-(pyrrolidin-1-yl)ethoxy] phenyl}-11H-indeno[1,2-c]quinolin-11-one
NSCLC: non-small cell lung cancer
CPT: camptothecin
DMSO: dimethyl sulphoxide
PI: propidium iodide
ECL: enhanced chemiluminescence
MMP: matrix metallopeptidase
PS: phosphatidylserine
RNase A: ribonuclease A
MAPK: mitogen-activated protein kinase
ERK: extracellular signal-regulated kinase
SDS-PAGE: SDS-polyacrylamide gel electrophoresis
SD: standard deviation
IC_50_: half maximal inhibitory concentration
