# Supplementary figures and images for: Dual roles of extracellular signal-regulated kinase (ERK) in quinoline compound BPIQ-induced apoptosis and anti-migration of human non-small cell lung cancer cells
Source: Cancer Cell Int. 2017 Mar 7;17:37. doi: 10.1186/s12935-017-0403-0 (PMC5339964; doi:10.1186/s12935-017-0403-0)

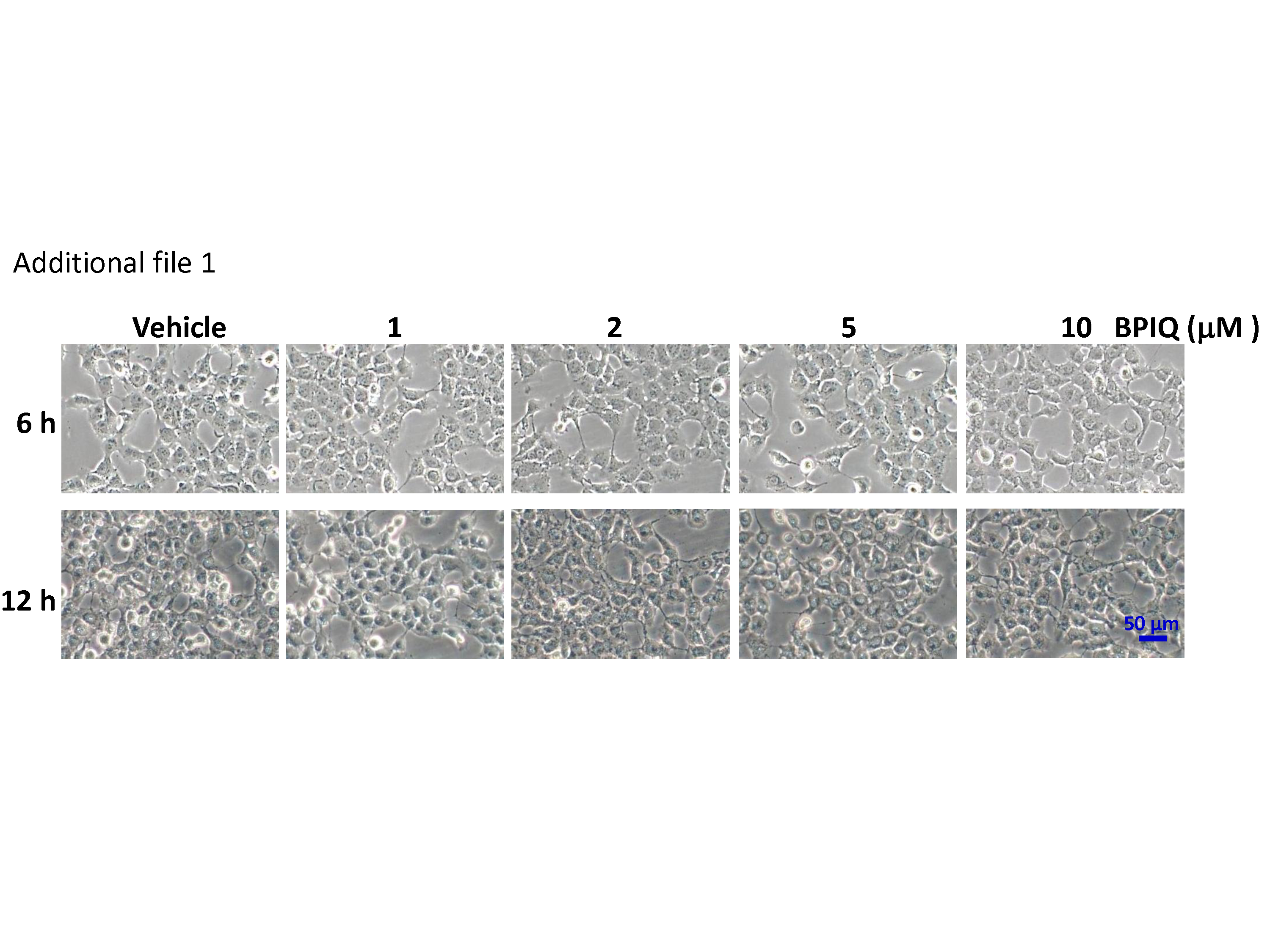

Supplement: Supplementary file 1 — Additional file 1. The effect of BPIQ on a time course of morphological changes of lung cancer cells. H1299 cells were treated with indicated concentrations of BPIQ for 6 and 12 h respectively. No significant changes of cellular morphology were observed. Magnification: 100×. [file 12935_2017_403_MOESM1_ESM.tiff]
